# *N*,*N*′-Bis(4-methyl­phen­yl)-*N*′′-(2,2,2-trichloro­acet­yl)phospho­ric triamide

**DOI:** 10.1107/S160053681202154X

**Published:** 2012-05-19

**Authors:** Akbar Raissi Shabari, Mehrdad Pourayoubi, Hassan Fadaei, Marek Nečas, Michal Babiak

**Affiliations:** a Faculty of Chemistry, North Tehran Branch, Islamic Azad University, Tehran, Iran; b Department of Chemistry, Ferdowsi University of Mashhad, Mashhad, Iran; c Department of Chemistry, Faculty of Science, Masaryk University, Kotlarska 2, Brno CZ-61137, Czech Republic

## Abstract

The P atom in the title compound, C_16_H_17_Cl_3_N_3_O_2_P, is bonded in a distorted tetra­hedral geometry with the phosphoryl and carbonyl groups *anti* with respect to one another. In the crystal, mol­ecules are linked through (N—H)_2_⋯O(=P) and N—H⋯O(=C) hydrogen bonds into chains along [001]. The phosphoryl O atom acts as a double hydrogen-bond acceptor.

## Related literature

For phospho­ric triamides having a C(=O)NHP(=O) skeleton, see: Pourayoubi *et al.* (2011 ▶). For the definition of a double hydrogen-bond acceptor, see: Steiner (2002 ▶); Pourayoubi *et al.* (2012 ▶).
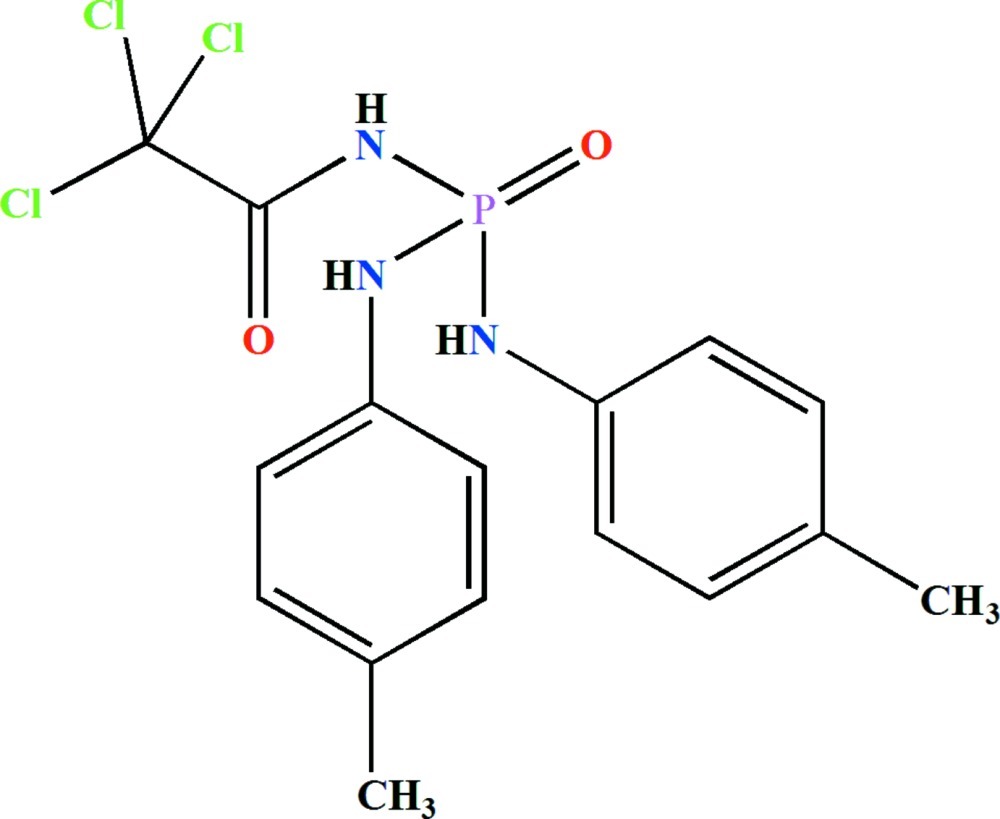


## Experimental

### 

#### Crystal data

C_16_H_17_Cl_3_N_3_O_2_P
*M* *_r_* = 420.65
Monoclinic, 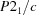

*a* = 17.5151 (6) Å
*b* = 10.8638 (4) Å
*c* = 9.8615 (3) Å
β = 97.565 (3)°
*V* = 1860.12 (11) Å^3^
*Z* = 4
Mo *K*α radiation
μ = 0.59 mm^−1^
*T* = 120 K
0.60 × 0.60 × 0.60 mm

#### Data collection

Oxford Diffraction Xcalibur Sapphire2 diffractometer
Absorption correction: multi-scan (*CrysAlis RED*; Oxford Diffraction, 2009 ▶) *T*_min_ = 0.955, *T*_max_ = 1.000
6796 measured reflections
3265 independent reflections
2820 reflections with *I* > 2σ(*I*)
*R*_int_ = 0.013

#### Refinement

*R*[*F* ^2^ > 2σ(*F* ^2^)] = 0.028
*wR*(*F* ^2^) = 0.072
*S* = 1.04
3265 reflections
240 parameters
H atoms treated by a mixture of independent and constrained refinement
Δρ_max_ = 0.33 e Å^−3^
Δρ_min_ = −0.26 e Å^−3^

### 

Data collection: *CrysAlis CCD* (Oxford Diffraction, 2009 ▶); cell refinement: *CrysAlis RED* (Oxford Diffraction, 2009 ▶); data reduction: *CrysAlis RED*; program(s) used to solve structure: *SHELXS97* (Sheldrick, 2008 ▶); program(s) used to refine structure: *SHELXL97* (Sheldrick, 2008 ▶); molecular graphics: *Mercury* (Macrae *et al.*, 2008 ▶); software used to prepare material for publication: *enCIFer* (Allen *et al.*, 2004) ▶.

## Supplementary Material

Crystal structure: contains datablock(s) I, global. DOI: 10.1107/S160053681202154X/lh5469sup1.cif

Structure factors: contains datablock(s) I. DOI: 10.1107/S160053681202154X/lh5469Isup2.hkl

Additional supplementary materials:  crystallographic information; 3D view; checkCIF report

## Figures and Tables

**Table 1:** Hydrogen-bond geometry (Å, °)

*D*—H⋯*A*	*D*—H	H⋯*A*	*D*⋯*A*	*D*—H⋯*A*
N1—H1*N*⋯O1^i^	0.77 (2)	2.17 (2)	2.8953 (19)	156 (2)
N2—H2*N*⋯O1^i^	0.76 (2)	2.23 (2)	2.948 (2)	159 (2)
N3—H3*N*⋯O2^ii^	0.75 (2)	2.31 (2)	3.008 (2)	157 (2)

Symmetry codes: (i) 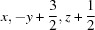; (ii) 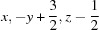.

**Fig. 1.**
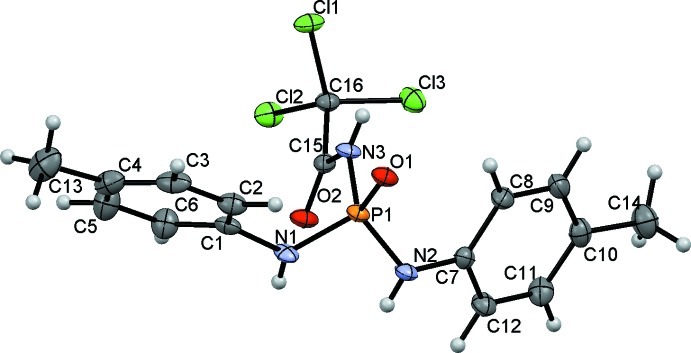
The molecular structure of the title compound with ellipsoids shown at the 50% probability level.

**Fig. 2.**
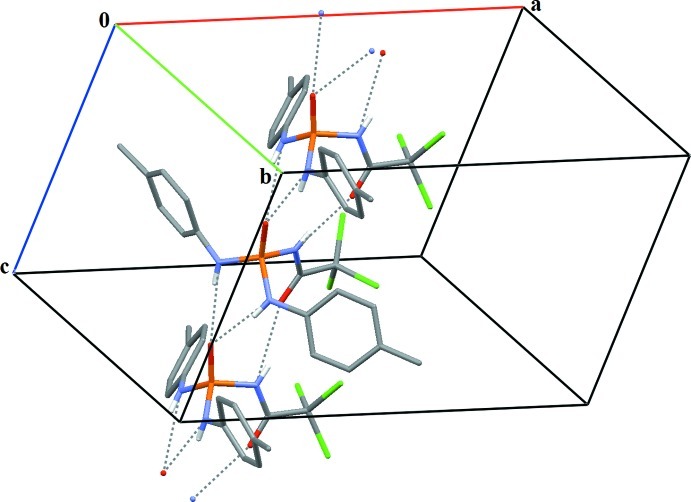
Partial packing view showing the formation of a chain through NC(O)NHP(O)—H···O(C) and (N—H)2···O(P) hydrogen bonds along the c axis. The dashed lines show the donor···acceptor distances of the hydrogen bonds.

## supplementary crystallographic information

### Comment

The structure determination of the title compound, P(O)[NHC(O)CCl_3_][NHC_6_H_4_(4-CH_3_)]_2_ (Fig. 1), was performed as a part of a project on the synthesis of new phosphoric triamides having a C(O)NHP(O) skeleton (Pourayoubi *et al.*, 2011).

The P═O (1.4727 (12) Å) and C═O (1.211 (2) Å) bond lengths are standard for this category of compounds (Pourayoubi *et al.*, 2011). The P atom has a distorted tetrahedral configuration (Fig. 1). The bond angles at the P atom are in the range 102.25 (8) – 118.28 (8)°. The P—N1 and P—N2 bonds (with lengths of 1.6195 (16) Å and 1.6345 (16) Å) are shorter than the P—N3 bond (1.7071 (16) Å). As might be expected the C15—N3 bond distance (1.349 (2) Å) is shorter than the other C—N bond distances.

In the crystal, each molecule is hydrogen-bonded to two adjacent molecules through N_C(O)NHP(O)_—H···O(C) and (N—H)_2_···O(P) hydrogen bonds along the *c* axis with the oxygen atom of phosphoryl group as a double-hydrogen bond acceptor (Steiner, 2002; Pourayoubi *et al.*, 2012).

### Experimental

CCl_3_C(O)NHP(O)Cl_2_ was synthesized from a reaction between phosphorus pentachloride (15.5 mmol) and 2,2,2-trichloroacetamide (15.5 mmol) in dry CCl_4_ at 353 K (3 h) and then treated with formic acid 85% (15.5 mmol) at ice bath temperature.

To a solution of CCl_3_C(O)NHP(O)Cl_2_ (1.7 mmol) in dry chloroform (30 ml), a solution of *p*-toluidine (6.8 mmol) in the same solvent (5 ml) was added at ice bath temperature. After 4 h stirring, the solvent was removed and the product was washed with distilled water and recrystallized from methanol at room temperature. IR (KBr, cm^-1^): 3305, 3248, 3029, 2920, 2858, 1714, 1619, 1514, 1433, 1376, 1277, 1234, 1191, 963, 882, 811, 730, 683.

Single crystals were obtained from a solution of the title compound in CH_3_OH after slow evaporation at room temperature.

### Refinement

All carbon bound H atoms were placed at calculated positions and were refined as riding with their *U*_iso_ set to either 1.2*U*_eq_ or 1.5*U*_eq_ (methyl) of the respective carrier atoms; in addition, the methyl H atoms were allowed to rotate about the C—C bond. Nitrogen bound H atoms were located in a difference Fourier map and refined isotropically.

### Crystal data

**Table:**

C_16_H_17_Cl_3_N_3_O_2_P	*F*(000) = 864
*M**_r_* = 420.65	*D*_x_ = 1.502 Mg m^−^^3^
Monoclinic, *P*2_1_/*c*	Mo *K*α radiation, λ = 0.71073 Å
Hall symbol: -P 2ybc	Cell parameters from 5060 reflections
*a* = 17.5151 (6) Å	θ = 3.3–27.7°
*b* = 10.8638 (4) Å	µ = 0.59 mm^−^^1^
*c* = 9.8615 (3) Å	*T* = 120 K
β = 97.565 (3)°	Prism, colourless
*V* = 1860.12 (11) Å^3^	0.60 × 0.60 × 0.60 mm
*Z* = 4	

### Data collection

**Table:**

Oxford Diffraction Xcalibur Sapphire2 diffractometer	3265 independent reflections
Radiation source: Enhance (Mo) X-ray Source	2820 reflections with *I* > 2σ(*I*)
Graphite monochromator	*R*_int_ = 0.013
Detector resolution: 8.4353 pixels mm^-1^	θ_max_ = 25.0°, θ_min_ = 3.5°
ω scan	*h* = −20→8
Absorption correction: multi-scan (*CrysAlis RED*; Oxford Diffraction, 2009)	*k* = −12→12
*T*_min_ = 0.955, *T*_max_ = 1.000	*l* = −11→11
6796 measured reflections	

### Refinement

**Table:**

Refinement on *F*^2^	Primary atom site location: structure-invariant direct methods
Least-squares matrix: full	Secondary atom site location: difference Fourier map
*R*[*F*^2^ > 2σ(*F*^2^)] = 0.028	Hydrogen site location: inferred from neighbouring sites
*wR*(*F*^2^) = 0.072	H atoms treated by a mixture of independent and constrained refinement
*S* = 1.04	*w* = 1/[σ^2^(*F*_o_^2^) + (0.0371*P*)^2^ + 0.8844*P*] where *P* = (*F*_o_^2^ + 2*F*_c_^2^)/3
3265 reflections	(Δ/σ)_max_ = 0.001
240 parameters	Δρ_max_ = 0.33 e Å^−^^3^
0 restraints	Δρ_min_ = −0.26 e Å^−^^3^

### Special details

**Table:**

Geometry. All e.s.d.'s (except the e.s.d. in the dihedral angle between two l.s. planes) are estimated using the full covariance matrix. The cell e.s.d.'s are taken into account individually in the estimation of e.s.d.'s in distances, angles and torsion angles; correlations between e.s.d.'s in cell parameters are only used when they are defined by crystal symmetry. An approximate (isotropic) treatment of cell e.s.d.'s is used for estimating e.s.d.'s involving l.s. planes.
Refinement. Refinement of *F*^2^ against ALL reflections. The weighted *R*-factor *wR* and goodness of fit *S* are based on *F*^2^, conventional *R*-factors *R* are based on *F*, with *F* set to zero for negative *F*^2^. The threshold expression of *F*^2^ > σ(*F*^2^) is used only for calculating *R*-factors(gt) *etc*. and is not relevant to the choice of reflections for refinement. *R*-factors based on *F*^2^ are statistically about twice as large as those based on *F*, and *R*- factors based on ALL data will be even larger.

### Fractional atomic coordinates and isotropic or equivalent isotropic displacement parameters (Å^2^)

**Table:**

	*x*	*y*	*z*	*U*_iso_*/*U*_eq_	
Cl1	0.42010 (3)	0.60382 (4)	0.43562 (4)	0.02457 (13)	
O1	0.14643 (7)	0.76151 (12)	0.35677 (12)	0.0207 (3)	
P1	0.17851 (3)	0.74597 (4)	0.50145 (4)	0.01634 (12)	
C1	0.12216 (10)	0.51775 (18)	0.53131 (17)	0.0191 (4)	
N1	0.13973 (9)	0.63843 (15)	0.58334 (15)	0.0190 (3)	
Cl2	0.46136 (3)	0.60149 (5)	0.72774 (4)	0.02712 (13)	
O2	0.30612 (7)	0.68782 (12)	0.72621 (12)	0.0225 (3)	
C2	0.07280 (11)	0.5003 (2)	0.41018 (18)	0.0246 (4)	
H2	0.0532	0.5692	0.3573	0.030*	
N2	0.17663 (9)	0.86377 (15)	0.60406 (16)	0.0187 (3)	
Cl3	0.44563 (3)	0.83378 (5)	0.58356 (5)	0.02685 (13)	
C3	0.05233 (11)	0.3820 (2)	0.3671 (2)	0.0287 (5)	
H3	0.0200	0.3709	0.2829	0.034*	
N3	0.27364 (9)	0.71385 (15)	0.49795 (15)	0.0182 (3)	
C4	0.07772 (11)	0.2797 (2)	0.4435 (2)	0.0292 (5)	
C5	0.12832 (12)	0.2987 (2)	0.5623 (2)	0.0335 (5)	
H5	0.1480	0.2298	0.6151	0.040*	
C6	0.15074 (11)	0.41629 (19)	0.6055 (2)	0.0276 (5)	
H6	0.1859	0.4270	0.6865	0.033*	
C7	0.22672 (10)	0.96637 (17)	0.60665 (17)	0.0180 (4)	
C8	0.25836 (11)	1.00236 (18)	0.49043 (18)	0.0228 (4)	
H8	0.2465	0.9582	0.4072	0.027*	
C9	0.30726 (12)	1.10297 (18)	0.4972 (2)	0.0258 (4)	
H9	0.3296	1.1253	0.4180	0.031*	
C10	0.32484 (11)	1.17239 (18)	0.61502 (19)	0.0250 (4)	
C11	0.29305 (12)	1.13365 (19)	0.7299 (2)	0.0280 (5)	
H11	0.3047	1.1781	0.8130	0.034*	
C12	0.24498 (11)	1.03244 (18)	0.72679 (18)	0.0237 (4)	
H12	0.2244	1.0081	0.8072	0.028*	
C13	0.05026 (13)	0.1515 (2)	0.4026 (3)	0.0414 (6)	
H13A	0.0835	0.0908	0.4550	0.062*	
H13B	0.0523	0.1396	0.3047	0.062*	
H13C	−0.0029	0.1410	0.4217	0.062*	
C14	0.37295 (13)	1.2873 (2)	0.6172 (2)	0.0350 (5)	
H14A	0.4040	1.2957	0.7069	0.052*	
H14B	0.3392	1.3590	0.5998	0.052*	
H14C	0.4071	1.2819	0.5462	0.052*	
C15	0.32379 (10)	0.69330 (17)	0.61173 (17)	0.0175 (4)	
C16	0.40963 (10)	0.68105 (17)	0.58949 (17)	0.0192 (4)	
H1N	0.1460 (12)	0.6449 (19)	0.662 (2)	0.023 (6)*	
H2N	0.1638 (12)	0.848 (2)	0.672 (2)	0.026 (6)*	
H3N	0.2878 (12)	0.720 (2)	0.430 (2)	0.027 (6)*	

### Atomic displacement parameters (Å^2^)

**Table:**

	*U*^11^	*U*^22^	*U*^33^	*U*^12^	*U*^13^	*U*^23^
Cl1	0.0236 (2)	0.0341 (3)	0.0165 (2)	0.0062 (2)	0.00442 (17)	−0.00528 (19)
O1	0.0204 (6)	0.0296 (7)	0.0124 (6)	−0.0004 (6)	0.0027 (5)	0.0013 (5)
P1	0.0169 (2)	0.0216 (3)	0.0107 (2)	−0.0005 (2)	0.00235 (17)	0.00029 (18)
C1	0.0155 (9)	0.0273 (10)	0.0154 (8)	−0.0044 (8)	0.0054 (7)	−0.0026 (8)
N1	0.0243 (8)	0.0253 (9)	0.0076 (7)	−0.0037 (7)	0.0025 (6)	−0.0019 (7)
Cl2	0.0263 (3)	0.0359 (3)	0.0180 (2)	0.0092 (2)	−0.00139 (18)	0.00249 (19)
O2	0.0227 (7)	0.0329 (8)	0.0125 (6)	0.0018 (6)	0.0047 (5)	0.0003 (5)
C2	0.0218 (10)	0.0349 (12)	0.0168 (9)	−0.0005 (9)	0.0010 (7)	−0.0019 (8)
N2	0.0224 (8)	0.0230 (9)	0.0118 (7)	−0.0006 (7)	0.0064 (6)	0.0011 (7)
Cl3	0.0271 (3)	0.0285 (3)	0.0252 (2)	−0.0063 (2)	0.00431 (19)	−0.00010 (19)
C3	0.0214 (10)	0.0447 (13)	0.0199 (10)	−0.0068 (10)	0.0015 (8)	−0.0129 (9)
N3	0.0196 (8)	0.0263 (9)	0.0099 (8)	0.0011 (7)	0.0059 (6)	0.0006 (6)
C4	0.0211 (10)	0.0318 (12)	0.0350 (11)	−0.0031 (9)	0.0050 (8)	−0.0110 (9)
C5	0.0296 (11)	0.0267 (11)	0.0411 (12)	−0.0014 (10)	−0.0065 (9)	−0.0007 (10)
C6	0.0271 (11)	0.0277 (11)	0.0254 (10)	−0.0017 (9)	−0.0062 (8)	−0.0013 (8)
C7	0.0173 (9)	0.0186 (9)	0.0177 (9)	0.0027 (8)	0.0011 (7)	0.0023 (7)
C8	0.0298 (10)	0.0225 (10)	0.0164 (9)	0.0016 (9)	0.0039 (7)	0.0014 (8)
C9	0.0287 (10)	0.0237 (11)	0.0262 (10)	0.0006 (9)	0.0080 (8)	0.0065 (8)
C10	0.0216 (10)	0.0229 (10)	0.0292 (10)	0.0010 (8)	−0.0018 (8)	0.0059 (8)
C11	0.0352 (11)	0.0249 (11)	0.0220 (10)	−0.0029 (9)	−0.0033 (8)	−0.0010 (8)
C12	0.0289 (10)	0.0259 (10)	0.0160 (9)	−0.0006 (9)	0.0025 (7)	0.0020 (8)
C13	0.0329 (12)	0.0363 (13)	0.0537 (15)	−0.0078 (11)	0.0003 (11)	−0.0157 (11)
C14	0.0323 (12)	0.0326 (12)	0.0386 (12)	−0.0074 (10)	−0.0010 (9)	0.0042 (10)
C15	0.0205 (9)	0.0174 (9)	0.0146 (9)	0.0002 (8)	0.0026 (7)	−0.0012 (7)
C16	0.0206 (9)	0.0226 (10)	0.0144 (9)	0.0007 (8)	0.0024 (7)	−0.0014 (7)

### Geometric parameters (Å, º)

**Table:**

Cl1—C16	1.7644 (18)	C5—C6	1.387 (3)
O1—P1	1.4727 (12)	C5—H5	0.9500
P1—N1	1.6195 (16)	C6—H6	0.9500
P1—N2	1.6345 (16)	C7—C12	1.386 (3)
P1—N3	1.7071 (16)	C7—C8	1.393 (2)
C1—C6	1.380 (3)	C8—C9	1.385 (3)
C1—C2	1.392 (2)	C8—H8	0.9500
C1—N1	1.427 (2)	C9—C10	1.385 (3)
N1—H1N	0.77 (2)	C9—H9	0.9500
Cl2—C16	1.7611 (18)	C10—C11	1.392 (3)
O2—C15	1.211 (2)	C10—C14	1.505 (3)
C2—C3	1.386 (3)	C11—C12	1.383 (3)
C2—H2	0.9500	C11—H11	0.9500
N2—C7	1.417 (2)	C12—H12	0.9500
N2—H2N	0.76 (2)	C13—H13A	0.9800
Cl3—C16	1.7786 (19)	C13—H13B	0.9800
C3—C4	1.383 (3)	C13—H13C	0.9800
C3—H3	0.9500	C14—H14A	0.9800
N3—C15	1.349 (2)	C14—H14B	0.9800
N3—H3N	0.75 (2)	C14—H14C	0.9800
C4—C5	1.388 (3)	C15—C16	1.553 (2)
C4—C13	1.510 (3)		
			
O1—P1—N1	115.74 (8)	C9—C8—C7	119.49 (18)
O1—P1—N2	118.28 (8)	C9—C8—H8	120.3
N1—P1—N2	102.25 (8)	C7—C8—H8	120.3
O1—P1—N3	104.64 (7)	C8—C9—C10	122.55 (18)
N1—P1—N3	109.71 (8)	C8—C9—H9	118.7
N2—P1—N3	105.77 (8)	C10—C9—H9	118.7
C6—C1—C2	119.16 (18)	C9—C10—C11	116.71 (18)
C6—C1—N1	119.83 (16)	C9—C10—C14	121.72 (18)
C2—C1—N1	120.91 (17)	C11—C10—C14	121.51 (18)
C1—N1—P1	124.64 (12)	C12—C11—C10	122.02 (18)
C1—N1—H1N	116.1 (16)	C12—C11—H11	119.0
P1—N1—H1N	114.9 (16)	C10—C11—H11	119.0
C3—C2—C1	119.68 (19)	C11—C12—C7	120.13 (17)
C3—C2—H2	120.2	C11—C12—H12	119.9
C1—C2—H2	120.2	C7—C12—H12	119.9
C7—N2—P1	124.35 (12)	C4—C13—H13A	109.5
C7—N2—H2N	114.8 (17)	C4—C13—H13B	109.5
P1—N2—H2N	113.8 (17)	H13A—C13—H13B	109.5
C4—C3—C2	121.81 (18)	C4—C13—H13C	109.5
C4—C3—H3	119.1	H13A—C13—H13C	109.5
C2—C3—H3	119.1	H13B—C13—H13C	109.5
C15—N3—P1	123.18 (13)	C10—C14—H14A	109.5
C15—N3—H3N	120.2 (17)	C10—C14—H14B	109.5
P1—N3—H3N	116.2 (17)	H14A—C14—H14B	109.5
C3—C4—C5	117.62 (19)	C10—C14—H14C	109.5
C3—C4—C13	121.82 (19)	H14A—C14—H14C	109.5
C5—C4—C13	120.5 (2)	H14B—C14—H14C	109.5
C6—C5—C4	121.3 (2)	O2—C15—N3	124.41 (16)
C6—C5—H5	119.3	O2—C15—C16	119.89 (15)
C4—C5—H5	119.3	N3—C15—C16	115.66 (14)
C1—C6—C5	120.31 (18)	C15—C16—Cl2	109.98 (12)
C1—C6—H6	119.8	C15—C16—Cl1	111.97 (12)
C5—C6—H6	119.8	Cl2—C16—Cl1	109.34 (10)
C12—C7—C8	119.06 (17)	C15—C16—Cl3	106.17 (12)
C12—C7—N2	119.70 (16)	Cl2—C16—Cl3	109.57 (10)
C8—C7—N2	121.24 (16)	Cl1—C16—Cl3	109.75 (9)
			
C6—C1—N1—P1	125.17 (17)	P1—N2—C7—C12	−152.11 (15)
C2—C1—N1—P1	−58.6 (2)	P1—N2—C7—C8	27.7 (2)
O1—P1—N1—C1	44.72 (17)	C12—C7—C8—C9	0.0 (3)
N2—P1—N1—C1	174.73 (14)	N2—C7—C8—C9	−179.82 (17)
N3—P1—N1—C1	−73.35 (16)	C7—C8—C9—C10	−1.7 (3)
C6—C1—C2—C3	0.9 (3)	C8—C9—C10—C11	2.3 (3)
N1—C1—C2—C3	−175.35 (16)	C8—C9—C10—C14	−174.99 (19)
O1—P1—N2—C7	−75.36 (16)	C9—C10—C11—C12	−1.3 (3)
N1—P1—N2—C7	156.22 (14)	C14—C10—C11—C12	176.03 (19)
N3—P1—N2—C7	41.39 (16)	C10—C11—C12—C7	−0.3 (3)
C1—C2—C3—C4	2.0 (3)	C8—C7—C12—C11	1.0 (3)
O1—P1—N3—C15	179.94 (15)	N2—C7—C12—C11	−179.19 (17)
N1—P1—N3—C15	−55.29 (17)	P1—N3—C15—O2	4.8 (3)
N2—P1—N3—C15	54.30 (17)	P1—N3—C15—C16	−172.98 (13)
C2—C3—C4—C5	−3.5 (3)	O2—C15—C16—Cl2	23.4 (2)
C2—C3—C4—C13	174.88 (19)	N3—C15—C16—Cl2	−158.68 (14)
C3—C4—C5—C6	2.1 (3)	O2—C15—C16—Cl1	145.21 (15)
C13—C4—C5—C6	−176.3 (2)	N3—C15—C16—Cl1	−36.9 (2)
C2—C1—C6—C5	−2.3 (3)	O2—C15—C16—Cl3	−95.03 (18)
N1—C1—C6—C5	174.01 (18)	N3—C15—C16—Cl3	82.86 (17)
C4—C5—C6—C1	0.8 (3)		

### Hydrogen-bond geometry (Å, º)

**Table:**

*D*—H···*A*	*D*—H	H···*A*	*D*···*A*	*D*—H···*A*
N1—H1*N*···O1^i^	0.77 (2)	2.17 (2)	2.8953 (19)	156 (2)
N2—H2*N*···O1^i^	0.76 (2)	2.23 (2)	2.948 (2)	159 (2)
N3—H3*N*···O2^ii^	0.75 (2)	2.31 (2)	3.008 (2)	157 (2)

Symmetry codes: (i) *x*, −*y*+3/2, *z*+1/2; (ii) *x*, −*y*+3/2, *z*−1/2.
